# Ultrasound-mediation of self-illuminating reporters improves imaging resolution in optically scattering media

**DOI:** 10.1364/BOE.9.001664

**Published:** 2018-03-13

**Authors:** Junaid Ahmad, Baptiste Jayet, Philip J. Hill, Melissa L. Mather, Hamid Dehghani, Stephen P. Morgan

**Affiliations:** 1 Optics and Photonics Research Group, Faculty of Engineering, University of Nottingham, University Park, NG7 2RD, UK; 2 Department of Electrical Engineering, University of Engineering and Technology, Lahore, KSK Campus, 54890, Pakistan; 3 School of Biosciences, University of Nottingham, LE12 5RD, UK; 4 Institute of Science and Technology in Medicine, Keele University, ST4 7QB, UK; 5 School of Computer Science, University of Birmingham, Edgbaston, B15 2TT, UK

**Keywords:** (170.1065) Acousto-optics, (110.0113) Imaging through turbid media, (110.7170) Ultrasound, (110.0110) Imaging systems, (040.1880) Detection, (290.4210) Multiple scattering

## Abstract

*In vivo* imaging of self-illuminating bio-and chemiluminescent reporters is used to observe the physiology of small animals. However, strong light scattering by biological tissues results in poor spatial resolution of the optical imaging, which also degrades the quantitative accuracy. To overcome this challenging problem, focused ultrasound is used to modulate the light from the reporter at the ultrasound frequency. This produces an ultrasound switchable light ‘beacon’ that reduces the influence of light scattering in order to improve spatial resolution. The experimental results demonstrate that apart from light modulation at the ultrasound frequency (AC signal at 3.5 MHz), ultrasound also increases the DC intensity of the reporters. This is shown to be due to a temperature rise caused by insonification that was minimized to be within acceptable mammalian tissue safety thresholds by adjusting the duty cycle of the ultrasound. Line scans of bio-and chemiluminescent objects embedded within a scattering medium were obtained using ultrasound modulated (AC) and ultrasound enhanced (DC) signals. Lateral resolution is improved by a factor of 12 and 7 respectively, as compared to conventional CCD imaging. Two chemiluminescent sources separated by ~10 mm at ~20 mm deep inside a 50 mm thick chicken breast have been successfully resolved with an average signal-to-noise ratio of approximately 8-10 dB.

## 1. Introduction

*In vivo* molecular imaging of bio - and chemiluminescent reporters is useful for a wide range of biological studies within the principles of humane experimental techniques. For example, to track progression of infectious diseases on the same cohort rather requiring groups of small animals (up to ten) to be sacrificed at defined time points and tissue removed for localized quantitative measurements (e.g. cell count, pathogen number) [1]. Bioluminescence (BL, biological enzymatic reaction) and chemiluminescence (CL, chemical reaction) are incoherent light sources, having similar underlying mechanisms for the production of light [2]. Although both BL and CL are useful, different BL assays have favorable pharmacokinetics that can exploit bioanalytical information of physiology [3]. Therefore, bioluminescence imaging (BLI) has gained much importance for being: sensitive; relatively low-cost; fast; non-invasive; and promising for the real-time imaging of tumors (up to several millimeters) inside small animals [4–8]. However, during *in vivo* imaging, light undergoes a strong scattering by the tissue components (such as membranes and different organelles) along its propagation path to the tissue surface that limits the performance of the current state-of-the-art optical imaging systems (e.g. Perkin Elmer, IVIS Spectrum *In Vivo* Imaging System) [9]. This reduces the image quality in terms of its spatial resolution and the ability of the measured intensity to be related to the concentration of the *in vivo* luminescent reporter [10].

One approach proposed to overcome the problem of light scattering is to introduce acoustic waves that propagate inside biological tissues with low scattering and attenuation. Light passing through the ultrasound (US) focus becomes modulated at the same US frequency and this ‘tagging’ can be used to localize the light sources deep inside tissue at an improved spatial resolution close to that of the US [11–13]. However, it is important to note that conventional acousto-optic sensing and tomography [14,15] uses coherent light (i.e. laser source) that produces a fluctuating speckle pattern at the surface of scattering medium. To address speckle decorrelation between successive measurements on the optical detection system various detection schemes have been introduced [16]. This work employs incoherent illumination where no speckle pattern is present and so detection can be performed using a single large area detector such as a photomultiplier tube.

Bioluminescence has virtually no background signal levels compared to those of fluorescent proteins where significant auto-fluorescence of the tissue surface results from the use of external excitation light sources [17,18]. It would therefore be advantageous if BLI could be combined with US modulation to improve the spatial resolution and reporter intensity quantification. In the past, Huynh et al. [19] have presented a proof of concept experiment to demonstrate this effect. The experiment used a low frequency 1 MHz US probe (that yields a ~4-5 mm wide acoustic focal zone), a relatively bright luminescent source in a tissue ‘like’ phantom having a very low optical scattering coefficient. The feasibility of translating this to *in vivo* imaging was later investigated in a numerical study using NIRFAST [20], an open source software tool that simulates the propagation of light in biological tissue based on finite element method (FEM). The mechanisms of ultrasound modulated bioluminescence tomography (USMBLT) [21] were investigated by modifying NIRFAST to incorporate the effects of US induced variations on the optical properties (scattering coefficient, absorption coefficient, and refractive index) of tissue and fluence rate from bioluminescence concentration located in a tissue sample. The simulation results showed US induced changes in BL concentration to produce a hundred times stronger modulation as compared to changes in the optical properties of the scattering medium. They inferred that it is feasible to detect US modulated bioluminescence signals having acceptable signal-to-noise ratio (SNR) when bioluminescence surface radiances are above 10^7^ photons/s/cm^2^/sr which is within the range of many *in vivo* experiments.

In view of the encouraging aforementioned USMBLT [21] simulation results, we have characterized the USMBLT system experimentally to investigate if the detection system is capable of imaging incoherent luminescent reporters with improved spatial resolution, and sufficient SNR. Preliminary results were presented in a recent conference paper [22]. In the research described here, bio-or chemiluminescent sources embedded inside a highly opaque scattering medium of known optical and acoustic properties were subjected to an acoustic wave in order to: (1) observe the US modulated light signals, and intensity of the luminescent sources; (2) generate acousto-optic 1D profiles that could be used localize multiple targets with improved spatial resolution. In contrast to previous research [19], the experiments presented here involve highly scattering thick tissue phantoms and chicken breast; and also utilises US frequencies and focal zones compatible with tissue imaging which helps to pave the way to *in vivo* imaging.

## 2. Materials and methods

### 2.1 Experimental configuration

**Fig. 1:**
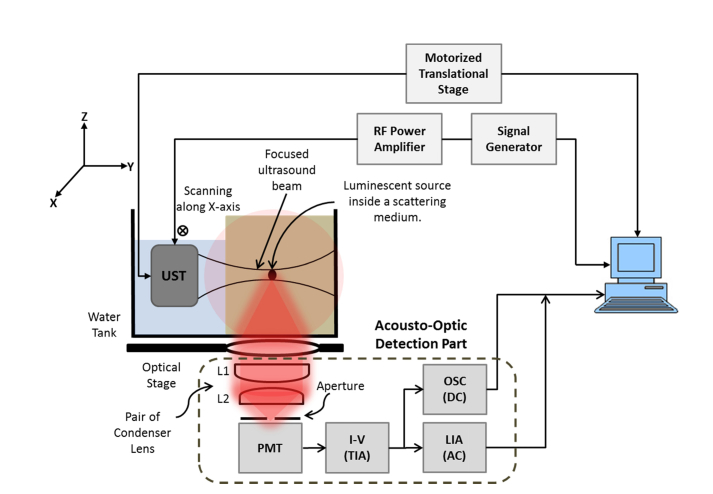
Schematic of acousto-optic platform for imaging incoherent low-level luminescent reporters (UST – ultrasound transducer, PMT – photomultiplier tube, TIA – transimpedance amplifier, LIA – lock-in amplifier, OSC – oscilloscope).

The experimental setup (Fig. 1

) comprises a signal generator (Tektronix AFG3022B) that supplies a continuous or quasi-continuous sinusoidal wave to an RF power amplifier (Amplifier Research 75A250A) to provide excitation to a focused US transducer (UST, Olympus Panametrics A380S-SU, 3.5 MHz central frequency, 25 mm diameter element, 49 mm focal length). This transducer is mounted on a computer-controlled translational stage (M-403.22S, Physik Instrumente) for the mechanical scanning of its focused acoustic beam to probe the scattering medium. A scattering phantom is submerged in an XYZ (120 mm × 200 mm × 100 mm) transparent Perspex water tank and the UST is situated within the tank at a distance ~49 mm (equal to the UST focal length) along the y-axis from the luminescent source embedded inside the scattering medium. Light from the surface of the scattering medium is collected from underneath the optical stage using a pair of aspherical condenser lens (L1 and L2, having respective diameter 75 mm and 50 mm; with a focal length of 50 mm and 37 mm, Edmund Optics Inc.) and a photomultiplier tube (PMT, H5783-20, Hamamatsu Photonics) with an aperture of diameter 8 mm placed ~80 mm from the water tank. The signals from the PMT are fed into a transimpedance amplifier (TIA, Stanford Research Systems SR445A, input impedance 50 Ω, −3 dB bandwidth 300 MHz), followed by a lock-in amplifier (LIA, Stanford Research Systems SR844, 12 dB/octave filter slope, and a full-scale sensitivity of 300 μV_rms_) that measures the acousto-optic modulation (AC signal) at the UST frequency, and an oscilloscope (OSC, Tektronix TDS2024B 8-bit ADC) to measure the total incident light (DC signal i.e. US enhanced, US modulated and unmodulated light). The LIA is synchronized by the UST drive signal as a reference. Before the experiment, the acoustic pressure generated by the UST has been measured using a hydrophone placed at the focal spot (Precision Acoustics, 1705 needle hydrophone). The entire experimental setup (Fig. 1) was placed in a dark room and housed in a light proof box for the PMT to minimize the background noise.

A LabVIEW-based interface was developed to control the movement of the translation stage that moves the UST along the x-axis and to record the ultrasound modulated (AC) and ultrasound enhanced (DC) signals at each position. The AC and DC line scans were produced by subtracting the noise floor voltages (of LIA and OSC, as a result of dark current and ambient light when the PMT is blocked) from the respective average values at each scan position. The recorded AC and DC average signals can be used to calculate the modulation depth AC/DC. The location of the light source within the scattering medium can be identified non-invasively by mapping the modulation depth of the US modulated optical signal at each scanning position. To calculate the SNR of the detected signal, the entire optical detection system (Fig. 1) has been modelled which enables us to calculate acousto-optically modulated AC current (I_AC_), the US enhanced signal (I_DC_), and the shot noise current (I_sht_):

$$I_{AC}=\frac{2V_{AC}}{Z_{TIA}G_{PMT}G_{TIA}}\left(A\right)$$ (2.1)

$$I_{DC}=\frac{V_{DC}}{Z_{TIA}G_{PMT}G_{TIA}}\left(A\right)$$ (2.2)

$$I_{sht}=\sqrt{2qI_{DC}B_{LIA}}\left(A\right)$$ (2.3)

$$SNR_{AC}(dB)=20\log_{10}\left(\frac{I_{AC}}{I_{sht}}\right)$$ (2.4)

$$SNR_{DC}(dB)=20\log_{10}\left(\frac{V_{DC}}{\sigma_{noise}}\right)$$ (2.5)

Where V_AC_ and V_DC_ are the modulated and total incident light signals at the LIA and OSC respectively. The other parameters in Eqs. (2.1-2.2-2.3) were obtained from the instruments datasheets as: PMT gain (G_PMT_ = 4 × 10^5^); gain of TIA (G_TIA_ = 5); TIA impedance (Z_TIA_ = 50 Ω); charge of an electron (q = 1.6 × 10^−19^ C); and equivalent noise bandwidth of the LIA (B_LIA_ = 1/8T, where T is the selected lock-in time constant corresponding to 12 dB/octave filter slope). The SNR_AC_ is calculated using Eq. (2.4) utilizing shot noise (I_sht_) and acousto**-**optically modulated current (I_AC_) from Eqs. (2.1) and (2.3). Whereas, SNR_DC_ for the DC signals in Eq. (2.5) simply uses V_DC_ and the standard deviation (σ_noise_) of the noise calculated from the data recorded by the oscilloscope. It is important to note that the above equations assume the system to be limited by the shot noise which is the optimum case and is observed in the experiments demonstrated.

### 2.2 Preparation of turbid media and luminescent sources

In small animal optical imaging, the intensity of exiting luminescence pattern formed at the surface of small animal (e.g. nude mouse) is a function of depth of the source (emitting at a particular wavelength) and *in vivo* light scattering [17]. Depending on the quantity and characteristics of different BL assays, it has been reported [23] that surface radiance of a nude mouse as recorded by the conventional pre-clinical optical imaging systems ranges from 10^5^ up to maximum of 10^11^ photons/s/cm^2^/sr. Using our system (Fig. 1) parameters, the surface radiance (L_DC_) of the turbid medium is calculated using the relationship [21]:

$$L_{DC}=\frac{V_{DC}}{h\nu S_{PMT}E_{Ano}Z_{TIA}G_{TIA}\Omega }$$ (2.6)

where h is Plank’s constant (6.634 × 10^−34^ J s), ν = c/λ is the light frequency, c is the speed of light (3 × 10^8^ m s^−1^), λ is the light wavelength, Ω is the collection solid angle, E_Ano_ and S_PMT_ is the anode radiance sensitivity and effective area of the PMT respectively.

**Fig. 2:**
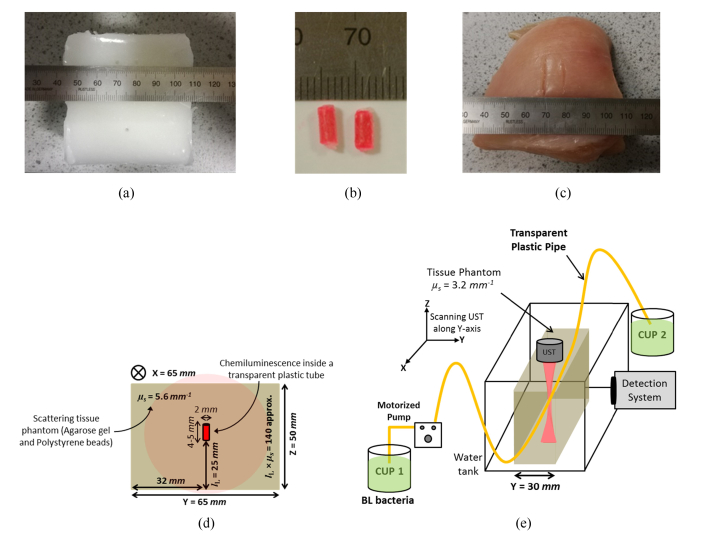
(a) Phantom gel with XYZ dimension 65 mm × 65 mm × 50 mm, scattering coefficient (µs) of 5.65 mm^−1^, g = 0.93; (b) Chemiluminescent sources encapsulated inside a FEP transparent tube using a parafilm; (c) Slab of a chicken breast; (d) Position of the source in the phantom and; (e) Schematic of the setup circulating glowing BL bacteria through the tissue phantom.

To perform the experiments using a scattering medium with a comparable surface radiance to *in vivo* imaging, tissue phantoms were prepared according to the procedure described elsewhere [24]. Briefly, agarose powder is dissolved in water that is stirred and heated, until it starts to boil. Afterwards, a specific quantity of polystyrene microspheres (proportional to optical scattering coefficient (μ_s_) ~5.65 mm^−1^, anisotropy factor g = 0.93) are dissolved in water using an ultrasonic bath which is eventually mixed with agarose solution without adding additional absorption so that the absorption coefficient is comparable to that of water. The resulting solution is cast, and solidified in a mould to form a tissue phantom as shown in Fig. 2(a)

. The phantom dimensions are 65 mm (X) × 65 mm (Y) × 50 mm (Z) and its acoustic properties (absorption coefficient is ~0.5 dB/cm MHz, when employing acoustic frequencies between 2 and 10 MHz) are comparable to that of soft tissue (absorption coefficient ~0.54 dB/cm MHz at 3.5 MHz [25,26]). To allow for a more realistic model, a slab of chicken breast having dimensions 70 mm (X) × 70 mm (Y) × 50 mm (Z) was also used to mimic an *in vivo* biological experiment as shown in Fig. 2(c). To generate luminescence inside the phantom gel, a chemiluminescent solution, with a central emission peak ~640 nm, is extracted from consumer market ‘glow sticks’. The CL material is enclosed using a parafilm in a light transparent Fluorinated Ethylene Propylene (FEP, Adtech Polymer Engineering Ltd.) tube having 0.2 mm wall thickness, inner diameter 2 mm, length ~4-5 mm, with a total volume of ~15 µL. This creates the light sources shown in Fig. 2(b) whose intensity decreases with a half-life of approximately 90 minutes. It should be noted that pouring the same quantity of CL directly into the tissue phantom results in rapid depletion of the light source (~10-15 minutes) due to interaction with agar gel (that is 98% water) which is inconvenient for the scanning experiments described here. Figure 2(d) presents a CL source implanted inside the phantom gel approximately at its center 32 mm (x and y-axis) with a depth 25 mm along z-axis, resulting in an optical thickness (l_L_ × µ_s_) i.e. product of the distance travelled by light from the luminescent source to the tissue phantom surface and scattering coefficient of ~140. This is comparable to a previously reported value of a mouse-shaped homogeneous tissue phantom used for the localization, and 3D reconstruction of bioluminescence sources [27]. The average surface radiance (L_DC_) from the prepared scattering phantom is calculated to be between 10^11^-10^12^ photons/s/cm^2^/sr using Eq. (2.6), which is comparable to the brightest BL assay used for conventional BLI.

To investigate BL, a strain of Photobacterium phosphoreum, with peak emission of 472 nm, isolated in the Indian Ocean was used. Bacteria were grown with shaking in instant ocean broth (instant ocean Aquarium systems, UK) 33 g/l, 5 g/l peptone, 3 g/l yeast extract; 22°C 200 rpm) until bioluminescence was induced [28]. Contrary to CL, it was not possible to encapsulate BL bacteria in the sealed FEP tube used for CL as they require oxygen to glow. In this case, a long transparent FEP pipe is embedded inside a tissue phantom having dimensions 80 mm (X) × 30 mm (Y) × 50 mm (Z) as shown in Fig. 2(e) at the time of casting. The emission wavelength and low surface radiance (~10^9^-10^10^ photons/s/cm^2^/sr) of the BL assay as compared to the CL reporter necessitated the use of a scattering phantom of lower optical thickness ~50. After the sample solidifies, the glowing BL bacteria (liquid) from one cup (CUP 1) is circulated at a constant flow rate through the pipe using a mini-variable pump motor and is subsequently collected by the receiving cup (CUP 2) as in Fig. 2(e). In this case, the UST is rotated by 90° for its easy advancement along Y-axis over the scattering phantom and the optical signals are detected from the front end of scattering medium rather than from the bottom.

### 2.3 Experiments

Experiments are carried out to investigate the effect of different parameters on the ultrasound modulated (AC) and ultrasound enhanced (DC) luminescence response in order to understand and optimize the detected signals. Firstly, the effect of acoustic pressure is investigated by aligning the US focal region with the CL target embedded in the tissue phantom with properties described in section 2.2. Continuous wave (CW) US is applied over the range 0-960 kPa in increments of ~96 kPa for a period of 150 s at each pressure and the AC signal is recorded on the LIA (time constant = 10 s) and the DC signal is recorded on the oscilloscope.

**Fig. 3:**
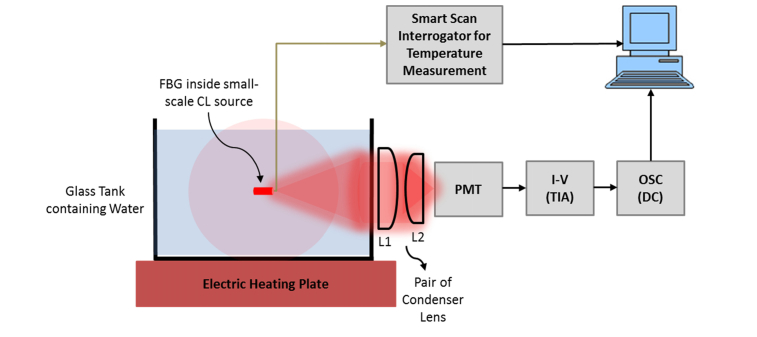
Heating water inside the glass tank to heat the small-scale CL source having 3 mm long FBG sensor inside the plastic tube to record the temperature and intensity of light using a PMT detector.

The use of CW US is likely to produce an increased DC CL signal due to either an increase in temperature [29,30] or an increase in the chemical reaction rate due to agitation of the sample [31]. The AC US signal will also increase due to these effects and due to the previously described US modulation methods [21]. Therefore, a second set of experiments is carried out to investigate and decouple these effects using a tissue phantom with the same properties as described in section 2.2. Temperature effects alone are isolated by removing the US transducer and heating the sample gently at 50°C for ~15 minutes using a heater plate (IKAMAG RET magnetic stirrer with heater, IKA Ltd., UK) as shown in Fig. 3

. Temperature was measured using an optical fibre sensor comprising a 3mm long Fibre Bragg Grating (FBG) manufactured and calibrated in-house [32]. The optical fibre sensor is thin (125 µm) and so can be easily position with the US focal zone close to the CL target. Wavelength shifts from the FBG were measured using an interrogator unit (Smart Scan Interrogator, Smart Fibres, UK) [33]. The manual heater plate is then replaced with an UST with its focal zone aligned with the CL target at an acoustic pressure of 960 kPa to investigate the equivalent change in the temperature.

A further set of experiments obtained 1D line scans of targets embedded within the tissue phantom and within a chicken breast. US line scans were carried across the scattering samples embedded with one or two luminescent targets to obtain AC and DC profiles. Two CL targets separated by ~10 mm were embedded at the mid-plane of a tissue phantom with properties described in section 2.2. Similarly, a piece of chicken breast was implanted with two CL targets separated by ~10 mm at the mid plane. The time taken by the system at each US scan location was 150 seconds. In case of the tissue phantom, 60 points over a 30 mm distance were recorded (step size 0.5 mm), which took 150 minutes. Whereas, when scanning the chicken breast only 40 points over the same distance (30 mm) were recorded, reducing the scan time to 100 minutes. At every scan point, the system acquires 50 data values of each AC and DC signals on the LIA (time constant = 1 s) and oscilloscope respectively. In order to compare with conventional optical imaging, a simple CCD camera (13 megapixel, f/2.0, autofocus, Huawei P9 lite camera) was also used to obtain 2D images that were converted to their respective 1D profiles. Finally, the effects of US mediation on BL has also been observed by circulating BL bacteria through the two transparent plastic FEP pipes separated by ~10 mm inside a tissue phantom.

## 3. Results

### 3.1 Influence of acoustic pressure on light source

Figure 4(a)

**Fig. 4:**
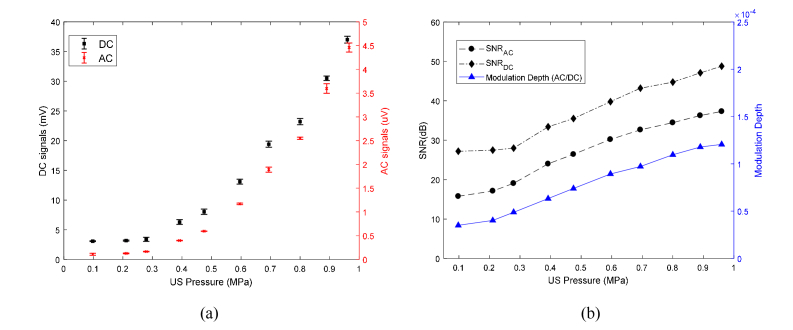
Influence of varying UST pressure on light source inside the phantom increases (a) Acousto-optic (AC) signals and intensity of light source (DC) and; (b) Modulation depth and SNR of the received AC and DC signals.

illustrates how an increase in pressure causes an increase in both the AC and DC signals whereas using the mechanisms described previously [21] relating to US modulation, only an increase in AC signal would be anticipated. The plot of SNR of AC and DC in Fig. 4(b) indicates the detectable signal levels for a range of pressures. Reasons for the increase in DC light level are investigated further in section 3.2, however, it is important to establish whether the increase in AC light level is due to an increase in broadband shot noise (Eq. 2.3) caused by the increase in DC light level or due to US modulation.

**Fig. 5:**
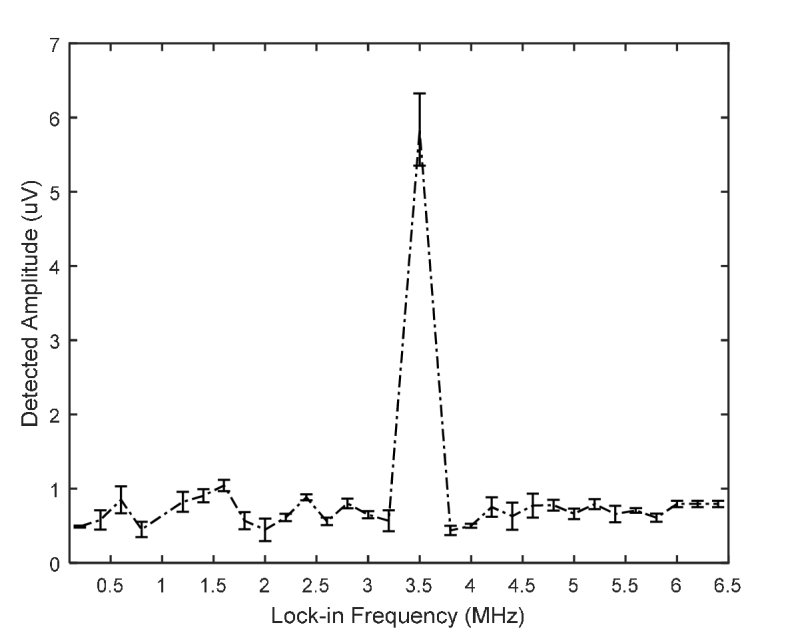
Amplitude of the detected AC signal vs reference frequency of the lock-in amplifier.

This was investigated by insonifying the same tissue phantom containing a newly prepared CL source with acoustic waves from the 3.5 MHz UST, and locking the LIA at different external frequencies (that are not integer multiples of 3.5 MHz) outside the US modulation frequency. Figure 5

presents AC signals along a frequency spectrum ranging from 0.2 to 6.4 MHz obtained by switching the LIA synchronizing reference signal at 0.2 MHz intervals while maintaining the US drive frequency at 3.5 MHz with acoustic pressure 960 kPa. If the AC signals were due to shot noise then Fig. 5 would be uniform along the frequency range but there is a clear peak at the US modulation frequency. This indicates that there is ultrasound modulation of the CL source in addition to a rise in the total light intensity.

### 3.2 Relationship between luminescence intensity and temperature rise

Figure 6(a)

**Fig. 6:**
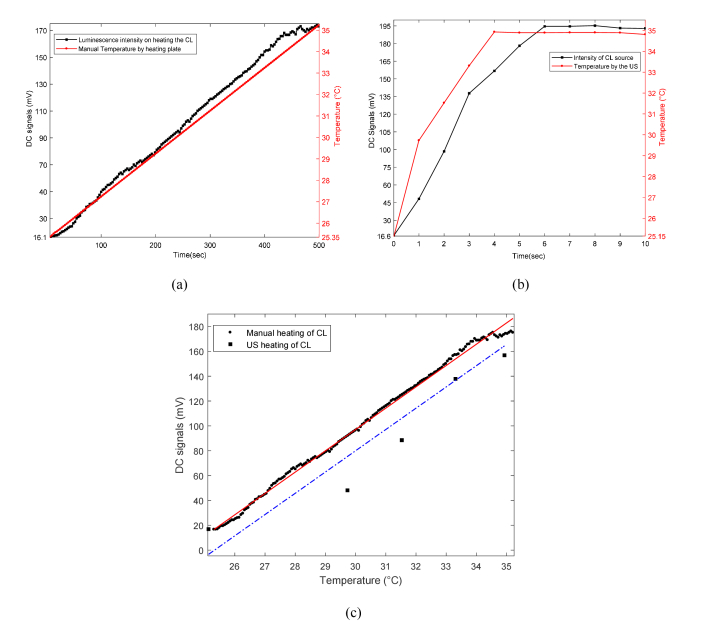
Relationship between CL intensity (DC) and temperature when: (a) heating the CL sample manually; (b) employing acoustic pressure 960 kPa from 3.5 MHz UST and; (c) fitting each of the two data sets (for manual and US heating) using linear regression to compare their respective slopes.

shows an increase in the detected optical intensity caused by increasing the temperature from room temperature using the system shown in Fig. 3. The temperature rises in an approximately linear way up to ~35.3°C (up to time = 500 s) with a corresponding increase in optical signals from the CL source but further heating results in a gradual decay (time > 500 s, not shown) in chemiluminescence. Replacing the heater plate with an UST focused on the CL target yields the results shown in Fig. 6(b). When the US is applied there is a rapid rise in temperature over first 5 s period and associated rise in the measured optical intensity. The temperature and DC intensity are both then maintained at ~34.8°C and ~195 mV respectively. The dominant effect causing the increase in DC intensity is clearly temperature. This has been justified using DC intensity and temperature values both from Fig. 6(a) for the manual heating and; Fig. 6(b) that is due to the US heating. Although a possible explanation of slight difference in DC intensities of Fig. 6(a) and (b) is likely due to using a fresh CL solution being used every time a new experiment is performed. This is confirmed by Fig. 6(c) which shows intensity vs temperature in both the cases (hot plate heating and US heating) and illustrates nearly the similar slopes for each of the two data sets.

**Fig. 7:**
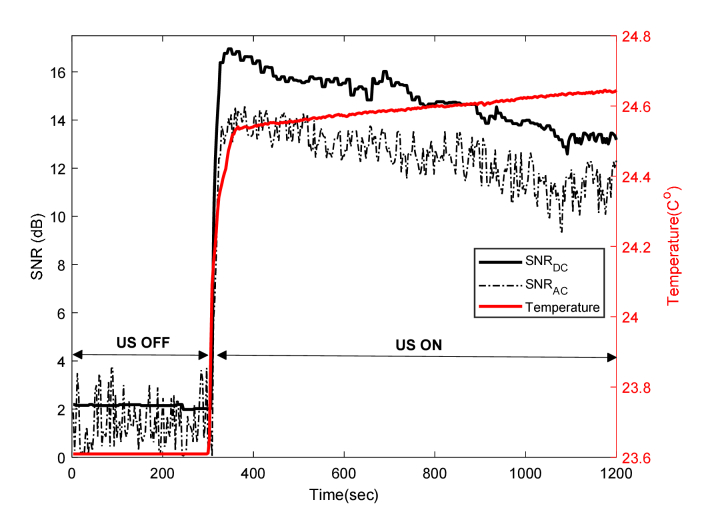
UST that uses 30% duty cycle of quasi-continuous acoustic bursts to mitigate the temperature rise up to ~1°C, while maintaining a significant increase in SNR for DC and AC from the CL source.

A temperature rise of 9-10°C is well above the mammalian tissue safety limits outlined by American Institute of Ultrasound in Medicine (AIUM) [34]. However, it should be noted that the rapid heating is largely due to the CL solution being held in a plastic tube as the temperature rise in the US focal zone positioned within an agarose gel phantom in a water bath without the plastic tube is only 2°C/hr. One method of maintaining the temperature at acceptable levels in the phantom experiments is to reduce the duty cycle of the US and a series of acousto-optic experiments were performed to determine an appropriate duty cycle, pressure and LIA time constant. An example is shown in Fig. 7

, where a 30% duty cycle of a quasi-continuous acoustic burst with a pressure of 960 kPa (1050 cycles, 300 µs time period, 1 ms trigger). The LIA time constant 100 ms was used, that is 100 times longer than the waveform trigger interval in order to provide a suitable time window for the LIA to detect sufficient, steady and stable AC signals. Figure 7 exhibits an initial temperature rise of ~1°C when switching the US ON (at time = 300 s) and a subsequent gradual increase. There is a corresponding increase in SNR of both DC and AC signals to detectable levels even after reducing the temperature rise to an acceptable safety limit for mammalian tissues. It is apparent from Fig. 7 that quasi-continuous US burst mitigate the large heating effects that reduce intensity of CL, thereby resulting in a lower SNR for DC that is comparable to the AC SNR. This is in contrast to Fig. 4(b) where the SNR difference between AC and DC is much larger. This is due to a significant rise in temperature because of the use of CW US at a pressure of 960 kPa.

### 3.3 US line scan imaging of turbid media implanted with luminescent targets

**Fig. 8:**
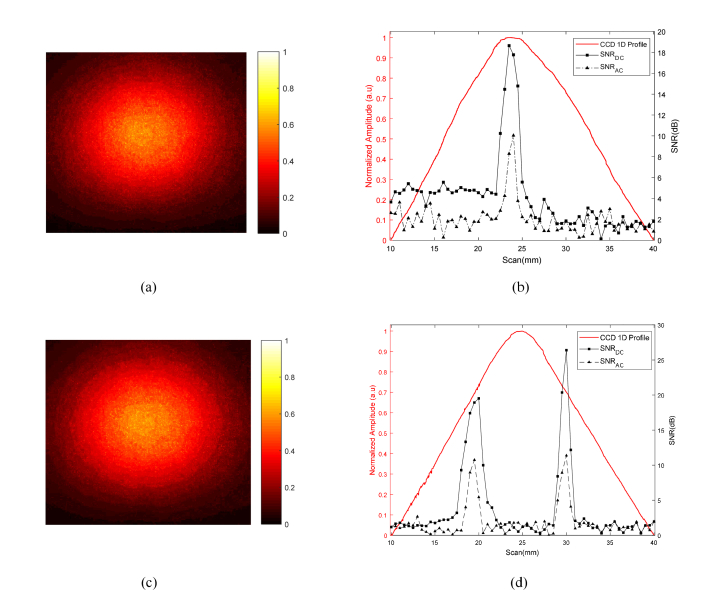
(a) Normalized CCD optical image of luminescence pattern formed at the surface of scattering tissue phantom for one CL target; (b) comparison of 1D cross-section of CCD image with 1D AC and DC profiles; (c) CCD image of two CL targets separated by ~10 mm implanted inside the turbid phantom and; (d) its respective 1D cross-section, AC and DC profiles.

**Fig. 9:**
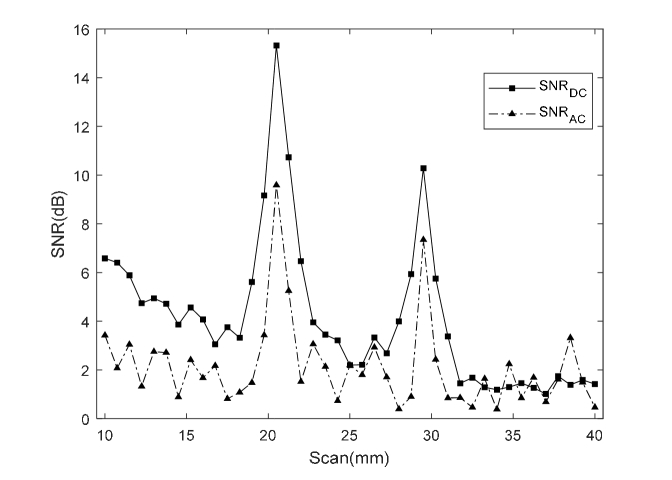
SNR traces of AC and DC, where UST scans (10 to 40 mm, step size = 0.75 mm) to resolve two CL targets separated by ~10 mm deep inside a slab of chicken breast.

**Fig. 10:**
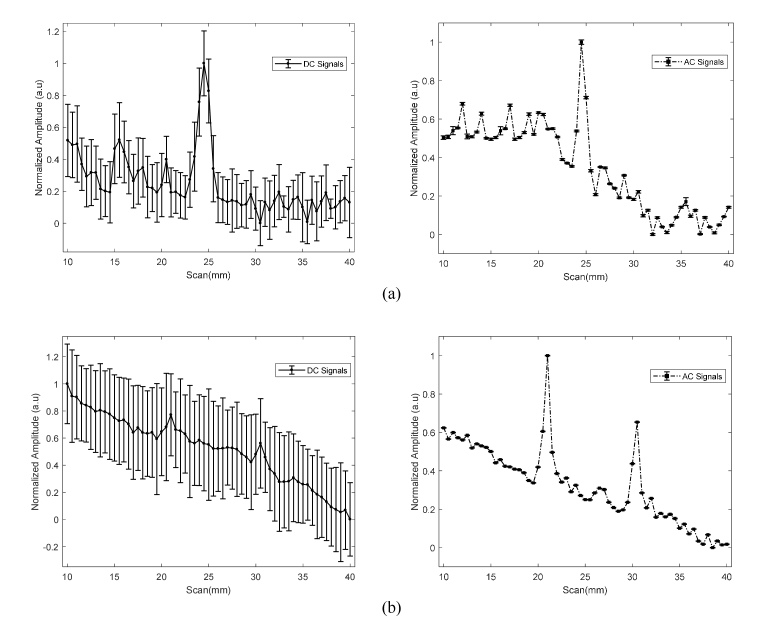
Acousto-optic system forming (a) DC and AC profiles resulting from circulation of BL assay through a single FEP plastic pipe passing through center of a tissue phantom and; (b) DC and AC signals for two FEP pipes separated by ~10 mm inside phantom.

In this section, US line-scan imaging has been performed using the optimized quasi-continuous acoustic burst parameters described in the previous section to maintain temperature rise within tissue safety limits for small animals. The 3.5 MHz UST is moved point-by-point over the scattering medium (with implanted luminescent targets) facing towards the acousto-optic detector as shown in Fig. 1. AC and DC data sets are acquired at each UST scan position and are used to generate the respective 1D profiles. To minimize the deviations in AC signals as much as possible, LIA time constant = 1 s is used to retain a sufficient quality of stable signals on the LIA even after reducing the temperature rise. Figure 8(a)

shows a normalized optical image from the CCD camera acquired before scanning. Figure 8(b) presents (i) a normalized 1D cross-section trace through the CCD image; (ii) 1D trace of DC (ultrasound enhanced) and; (iii) AC in terms of their respective SNR. The UST scans from 10 to 40 mm with a step size of 0.5 mm over the tissue phantom having one source target placed at a scan position of ~23 mm. The improvement in lateral resolution is apparent from Fig. 8(b), where AC and DC traces show full width half maximum (FWHM) recovered using Gaussian curve fitting to be 1.3 mm and 2.3 mm respectively, compared to the conventional CCD image trace (FWHM = 16 mm). On the other hand, the CCD image in Fig. 8(c) and its 1D cross-section in Fig. 8(d) shows a single maximum for the two luminescent targets inside the scattering medium which makes it difficult to observe the existence of multiple targets, unlike the respective SNR traces of AC and DC. This clearly displays the benefit of combining US with light that helps resolve and locate two CL targets with a peak separation of 10.5 mm. In addition to this, the acoustic-optic system enables imaging of two CL targets inside a slab of chicken breast (Fig. 9

) with spatial resolution comparable to that shown in Fig. 8. For BL assay (Fig. 2e), steering the US through the scattering gel with a plastic pipe circulating the BL bacteria demonstrates that the targets can be localized (Fig. 10 (a)

). Figure 10(b) presents US line-scans where two plastic tubes circulating BL through the tissue phantom are separated by a peak difference ~10.5 mm.

## 4. Discussion

The results in Fig. 4 demonstrated US modulation (AC) of CL within the focused acoustic beam within a heavily scattering medium with a modulation depth and SNR that indicates the potential for *in vivo* imaging. However, the increase in luminescence intensity (DC) was not predicted in the numerical model by Zhang et al. [21] and warranted further investigation. Two mechanisms have previously been proposed for increases in DC intensity due to US: sonochemical increase in chemical reaction rate [31] and temperature [35]. The experiments performed in section 3.2 clearly illustrate the rise in temperature to be dominant factor increasing the DC signals. Zhao et al. [35] reported a red-shift and significant increase in the intensity of bioluminescence (i.e. firefly luciferase) spectra with temperature (rising from 25 to 37°C). This mechanism was referred as ‘temperature-modulated’ bioluminescence tomography (BLT) by Wang et al. [36]. They performed simulations in which an array of US transducers was used to heat the volume of interest sequentially to induce detectable optical changes at the mouse surface. This can be used to overcome the inherent illposedness of BLT. However, until now this has not been demonstrated experimentally.

The increase in DC light level is useful as it can be implemented as part of a conventional CCD camera imaging system, rather using lock-in detection in parallel to observe the AC. The increase in AC (Fig. 5) is due to ultrasound modulation rather than increased shot noise because it is bigger at the modulation frequency of the UST. The effects of temperature rise by the US (acoustic absorption by plastic tube) can be kept within safety limits by changing the acoustic duty cycle. The probability of adverse bioeffects depends on both the duration of US exposure and magnitude of temperature rise. According to AIUM [34], acoustic heating inducing a temperature elevation less than or equal to 1.5°C does not produce harmful bioeffects to mammalian tissues for an US exposure of 50 hours. In practice, of course, BL/CL reporters would not be contained within a plastic tube and so heating effects to harmful levels would be much smaller.

Figure 8 presents a comparison between the 1D optical images formed by the conventional CCD camera for one or more CL targets implanted inside a scattering tissue phantom; with their respective AC and DC images generated by the mediation from UST. It was observed in Fig. 8(c and d) that the CCD image forms a single maximum compared to the ultrasound modulated (AC) and ultrasound enhanced (DC) images in which two luminescent targets can be located. It is worth explaining here that scan results in Fig. 8(b) show a slight decay in the CL intensity which is not observed in Fig. 8(d). This is due to the fact that both experiments were performed at different times with newly prepared CL sources which varies the light intensity detected by the PMT each time new experiment is performed as discussed in section 3.2. Figure 8(d) also shows a small difference in the increased intensity of two CL sources which is due to slightly uneven positioning of the sources inside the tissue phantom with respect to the focused US beam. This has also been demonstrated in a chicken breast that is both scattering and absorbing. In the results (Fig. 8), the FWHM of the DC profile is slightly larger than the AC profile. When the US focal zone approaches the luminescent target (in its vicinity, not necessary overlapping), the ultrasonic field is absorbed by the plastic tube that holds the target and the DC signal increases due to heating. However, the AC signal is only observed when the US focal zone overlaps the source target which results in a smaller FWHM.

Using the CL reporter in section 3.1-3.3 provides more stable results due to a relatively constant and slow decay characteristic of CL. The DC light enhancement is also relatively high as the CL target is housed in a plastic tube which facilitates an increase in temperature. For BL, the photobacterium phosphoreum assay used in experiments is much less bright than CL and its intensity varies with its circulation inside the pipe and exposure to the environment when collecting in a cup. In this case, the DC enhancement is lower because the heat generated by the ultrasound is rapidly dissipated by the flows through the tube. This is apparent in Fig. 10 that shows large variations (error bars) in the DC intensity from BL assay. Both AC and DC signals were normalized and it was interesting to observe a lower but stable AC modulation. However, the intensity of the BL marine bacteria used in our experiments decays more rapidly than CL, and they require oxygen supply for them to remain active. The BL assays are easy to use but are not optimized for *in vivo* imaging. However, considerable efforts are currently underway to establish novel luciferases that can exerts a stable, brighter, and red-shifted bioluminescence by mutation [37,38]. The lateral resolution is observed to be of the order of a few mm, which is close to the theoretical value (size of the ultrasound focal spot ~1.33 mm). In future higher frequencies and pulsed ultrasound can be used to improve spatial resolution further in lateral and depth directions. There will of course be a trade-off in SNR when higher frequencies or pulsed US is used [39–41].

To our knowledge, this is the first experimental demonstration of US mediation of bioluminescent reporters in thick tissue phantoms. The consideration and understanding of temperature effects is an important step forward in translating this approach into biomedical application. However, the system is not devised to acquire images in 3D and also the scan time is currently long and is unlikely to be practical for small animal imaging. In future this can be improved by using multiple US transducers arranged in a ring around the animal which is often used in photoacoustic imaging; or by moving to a phased array transducer; or by reducing the signal acquisition time at each US scan point or by employing pulsed ultrasound (albeit at the expense of SNR). The reduction of SNR could be compensated for with brighter BL assays.

## 5. Conclusions

An acousto-optic system to image low intensity BL and CL reporters embedded inside a scattering medium has been demonstrated. In general, the acoustic waves from an UST interact with the target reporters implanted inside the scattering medium and this can be used to improve spatial resolution of optical imaging. Results have demonstrated feasibility in scattering phantoms comparable to that of small animal imaging (agar gel phantoms and chicken breast). Two effects are observed that can be used to improve spatial resolution: (i) enhancement of the total (DC) intensity due to US; (ii) US modulation (AC) of the luminescence. Further investigation of the origin of (i) demonstrates that this due to an increase in temperature rather than an increase in the chemical reaction rate and we believe that this is the first experimental demonstration of temperature modulated bioluminescence imaging. DC enhancement is beneficial as it could easily be used as a part of conventional optical imaging system (such as IVIS [9]). Temperature increases can be maintained within safe limits by varying the acoustic duty cycle. This technique has potential for its in-conjunction use with existing state-of-the-art small animal (i.e. nude mouse) imaging system. Ultrasound modulated (AC) imaging utilises more complex instrumentation (lock-in detection and scanning) than a CCD but the AC signals demonstrate spatial resolution close to that of the US frequency and more robust signals for imaging bioluminescence. In future, this research has the potential to contribute to the 3Rs [42]. Replacement: better imaging will inform more accurate computational models; Reduction: imaging enables longitudinal studies on the same cohort, more accurate quantitative imaging allows fewer animals to be used in a study; Refinement: through the improved quality of research findings.
